# Drug Reaction With Eosinophilia and Systemic Symptom (DRESS) Following Rifampicin Treatment: A Case Report

**DOI:** 10.7759/cureus.19223

**Published:** 2021-11-03

**Authors:** Ramesh Shrestha, Shivendra K Jha, Jasmine Bartaula

**Affiliations:** 1 Infectious Disease, Sukraraj Tropical and Infectious Disease Hospital, Kathmandu, NPL; 2 Dermatology, Venerology and Leprology, Sukraraj Tropical and Infectious Disease Hospital, Kathmandu, NPL; 3 Infectious Disease, Chitwan Medical College, Tribhuvan University, Chitwan, NPL

**Keywords:** rifampicin, tuberculosis, dress, type iv hypersensitivity, dermatology, immunology, allergy, anti-tubercular drug

## Abstract

Drug reaction with eosinophilia and systemic symptoms (DRESS) is an idiosyncratic severe cutaneous adverse reaction (SCAR) characterized by a skin rash with systemic involvement (e.g., hematological, solid organ abnormalities). Various medications, most commonly anticonvulsants (carbamazepine, phenytoin), antibiotics (vancomycin, amoxicillin), and sulfa drugs (dapsone, sulfasalazine), have been implicated. We report a case of a 75-year-old man with pulmonary tuberculosis under anti-tubercular treatment (ATT Category 1 as per the national guidelines of Nepal) presenting with rash, fever, liver dysfunction, and eosinophilia, a combination of features suggestive of DRESS. According to the national tuberculosis (TB) survey of 2018-2019, over 117,000 people in Nepal were living with TB, including 69,000 newly diagnosed people. In third-world countries, such as Nepal, with a high TB prevalence, and the Southeast Asian region (with a huge percentage of the global burden of ‎TB‎ incidence), the risk of life-threatening adverse drug reactions during ATT is high. However, a good response is seen if it is recognized early and on stopping ATT and receiving a course of steroids and emollients.

## Introduction

Drug reaction with eosinophilia and systemic symptoms (DRESS) is also termed interchangeably as drug-induced delayed multi-organ hypersensitivity syndrome (DIDMOHS) and drug-induced hypersensitivity syndrome (DIHS) [1]. ⁠It likely involves drug-induced herpesvirus reactivation followed by clonal expansion of T-cells, which cross-react with the medication. It is a delayed type-IV hypersensitivity reaction, which is often life-threatening [2]. However, the immune response and the clinical significance of T-cell receptor (TCR) repertoire reformation during the course of DRESS are not known [3]. ⁠Various medications, most commonly anticonvulsants (carbamazepine, phenytoin), antibiotics (vancomycin, amoxicillin), and sulfa drugs (dapsone, sulfasalazine), have been implicated [4].⁠ DRESS describes a severe medication-induced adverse reaction with cutaneous, hematological, and solid-organ features [2,4-5]. In cutaneous manifestation, facial edema is conspicuous along with a generalized papulopustular or exanthematous rash, which may evolve to exfoliative dermatitis. Systemic manifestations include lymphadenopathy, hematological abnormalities (hypereosinophilia and the presence of atypical lymphocytes or mononucleosis), and organ involvement such as hepatitis, nephritis, pneumonitis, myocarditis, hypothyroidism, and encephalitis, occurring after three to six weeks of drug therapy [4]. ⁠This response results in death 10% of the time [2,4].

## Case presentation

**Figure 1:**
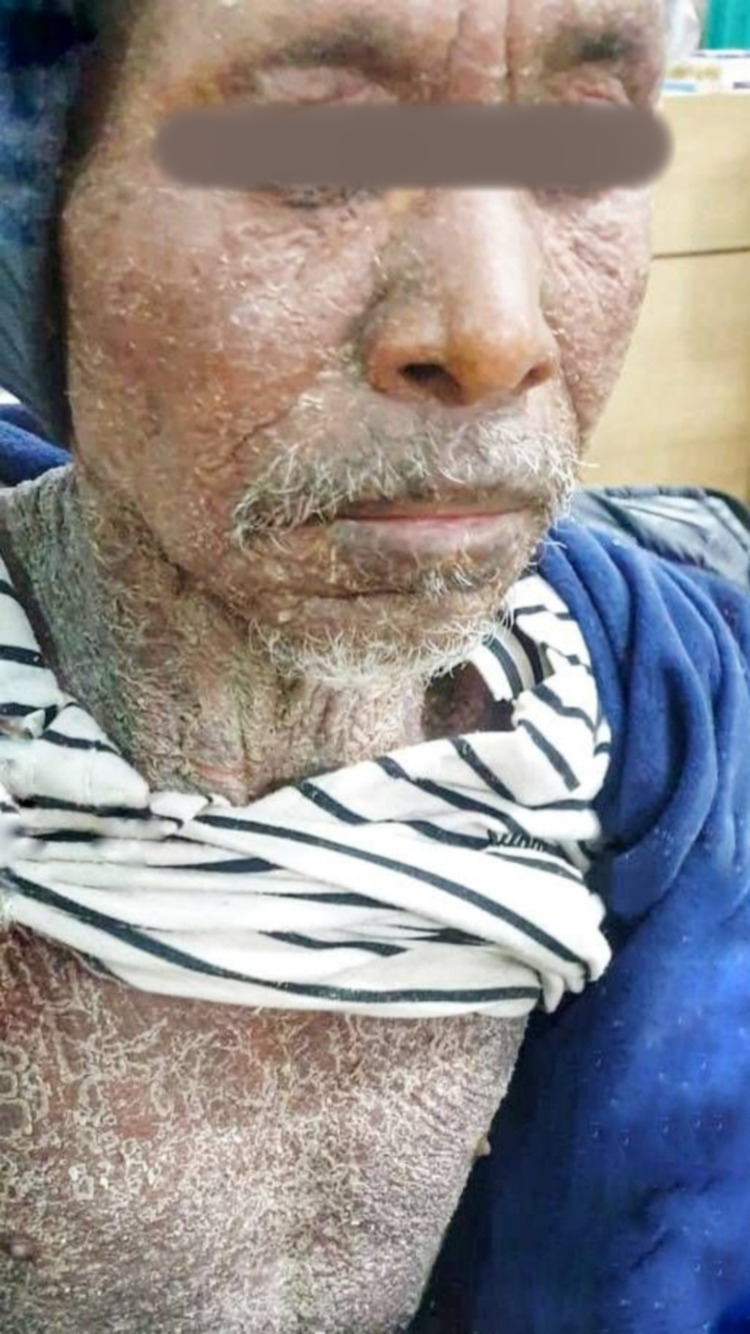
Clinical manifestations involving the face and trunk

**Figure 2:**
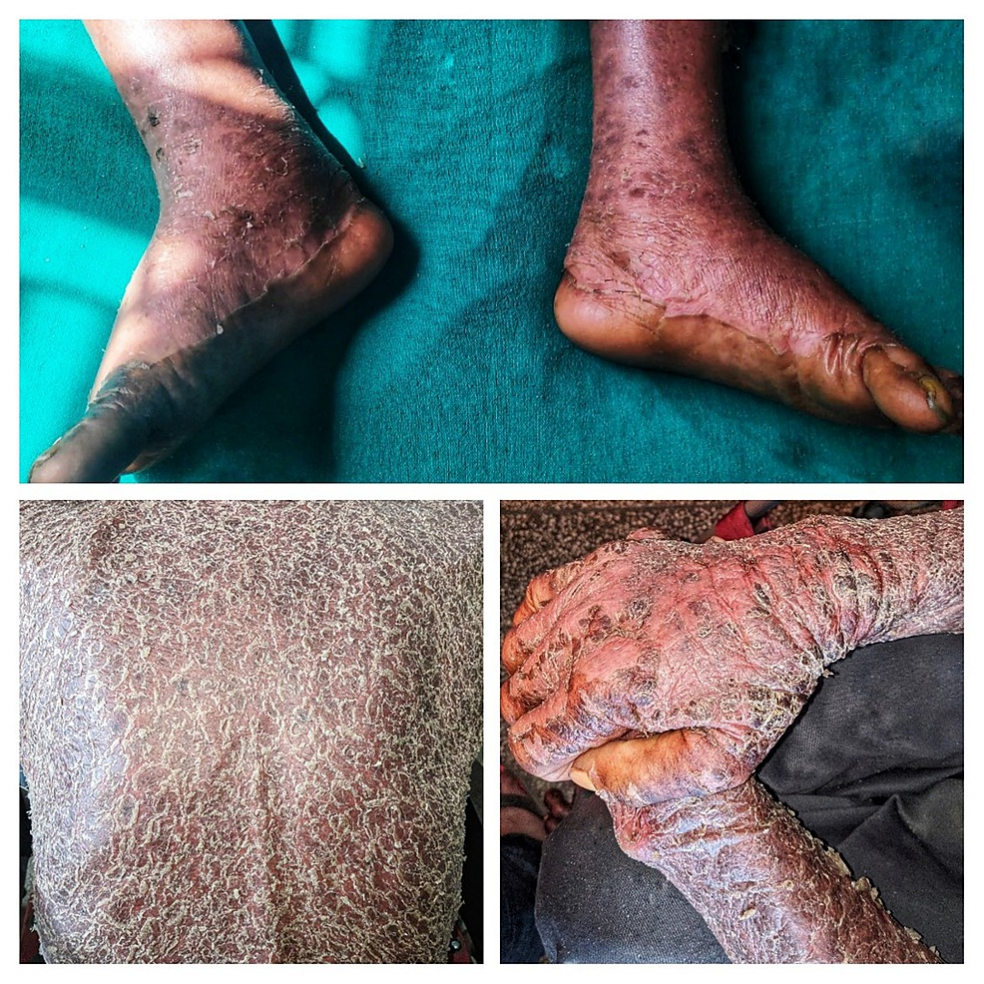
Cutaneous manifestation of drug hypersensitivity

A 75-year-old male, a known case of chronic obstructive pulmonary disease (COPD), presented with whitish, coarse, thick, exfoliative scaling throughout the body. Three months prior, he had been diagnosed with pulmonary tuberculosis and, at the time of presentation, was under antitubercular therapy (ATT) 2HRZE/4HR CAT1 as per Nepal guidelines [6]. When the patient was in the continuation phase of antitubercular therapy, i.e., isoniazid (H) and rifampicin (R), he gave a history of itching throughout the body, which was acute in onset, initially localized over the trunk and bilateral proximal extremities, with no diurnal variation, severe enough to disturb his sleep pattern, with no aggravating factor and a mild improvement on taking antihistaminic medication. The itching gradually progressed to the face, distal extremities, and upper and lower back over 15 days. It was accompanied by a maculopapular rash that initially consisted of multiple, localized lower extremity and trunk lesions and later became confluent, diffuse, and gradually involved the entire body. There were palpable, blanching, erythematous papules and a confluent of plaques involving 90% of the body surface area, symmetrically distributed throughout the body, but sparing the genitalia, associated with the thick, coarse, semi-adherent scale-like overlying plaques (Figures 1-2). The patient reported soreness with swallowing that started 15 days prior. There were moist mucous membranes; slightly edematous, dry, cracked lips; and oral mucosa ulcers, which started three to four days prior.

At presentation, he had facial and bilateral non-pitting pedal edema (Figures 1-2) and showed respiratory distress with cough.

He had a fever of 102^o^F, a heart rate of 120 beats/min, blood pressure of 125/70 mmHg, was tachypneic, and had yellowish discoloration of sclera and oxygen saturation of 64% on room air and 94% on 6L of oxygen. He had tender, significant enlarged lymph nodes at the axillary, cervical, and inguinal regions. He was alert and oriented. However, he had respiratory distress and a cough with bilateral wheeze. The cardiovascular, neuromuscular, gastrointestinal, and locomotor examination findings were unremarkable.

Dermatology and Allergy/Immunology were consulted. ATT was immediately discontinued, and intravenous fluid was started with oral protein supplementation. He received broad-spectrum intravenous antibiotics. The prolonged latency period of more than two months and temporal association of symptoms after starting ATT led to possible drug reactions. The diagnosis was made based on clinical grounds and RegiScar guidelines. The other differentials were Steven Johnson syndrome (SJS), mycosis fungoids, maculopapular rash secondary to infections, acute lupus erythematosus, and erythroderma.

**Figure 3:**
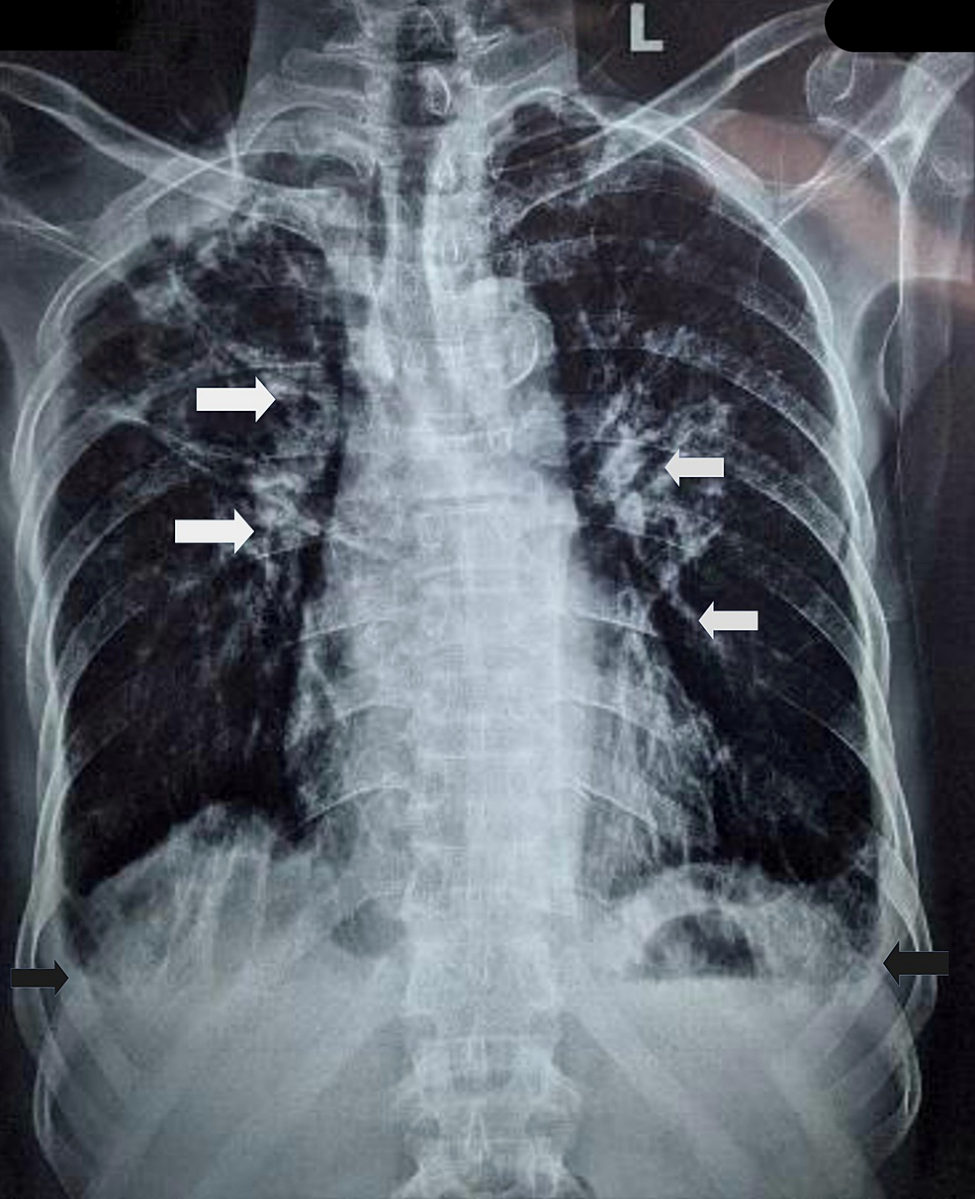
Chest X-ray PA view Few patchy areas of ground-glass opacities in B/L lungs; mild B/L pleural effusions (black arrow); calcified mediastinal and hilar lymphadenopathy (white arrow) PA: posteroanterior; B/L: bilateral

The patient's laboratory results are shown in Table 1, demonstrating leukocytosis with eosinophilia of 60% and absolute eosinophil count of 10.4x10^9^/L, peripheral blood smear showing atypical lymphocytes along with deranged liver function suggestive of systemic involvement. The chest X-ray (Figure 3) showed multiple nodules in bilateral lungs; small cavitary lesions in the right upper lobe and left lower lobe; few patchy areas of ground-glass opacities in bilateral lungs; mild bilateral pleural effusion; calcified mediastinal; and hilar lymphadenopathy suggestive of COPD superimposed with pneumonitis.

**Table 1:** Laboratory parameters of the patient from admission to recovery TLC: total leukocyte count; /Cu mm: per cubic millimeter; N: neutrophil; L: lymphocyte; E: eosinophil; M: monocyte; ALP: alkaline phosphatase; SGPT: serum glutamic pyruvic transaminase; SGOT: serum glutamic oxaloacetic transaminase

Lab Parameters	Day 1	Day 3	Day 5	Day 6	Day 10	Day 11	Day 14	Day 21
TLC (/mm^3^)	17,300	17,300	11,000	9,300	6,300	6,500		5,300
N (%)	22	22	80	57	78	78		40
L (%)	10	10	11	10	16	16		38
E (%)	60	60	50	27	03	03		05
M (%)	8	8	4	10	03	03		17
Urea (mg/dl)		43	45			57	57	35
Creatine (mg %)		0.5	0.6			0.7	0.7	0.8
Sodium (mEq/L)		140	136			138	138	134
Potassium (mEq/L)		3.8	5.2			4.4	4.2	3.9
Bilirubin T (mg/dl)	8.4	7.8	5.5			1.7		0.7
Bilirubin D (mg/dl)	2.8	2.5	1.3			0.3		0.2
ALP (U/L)	240	236	234			217		187
SGPT (U/L)	319	224	132			67		69
SGOT (U/L)	275	254	169			52		54

**Figure 4:**
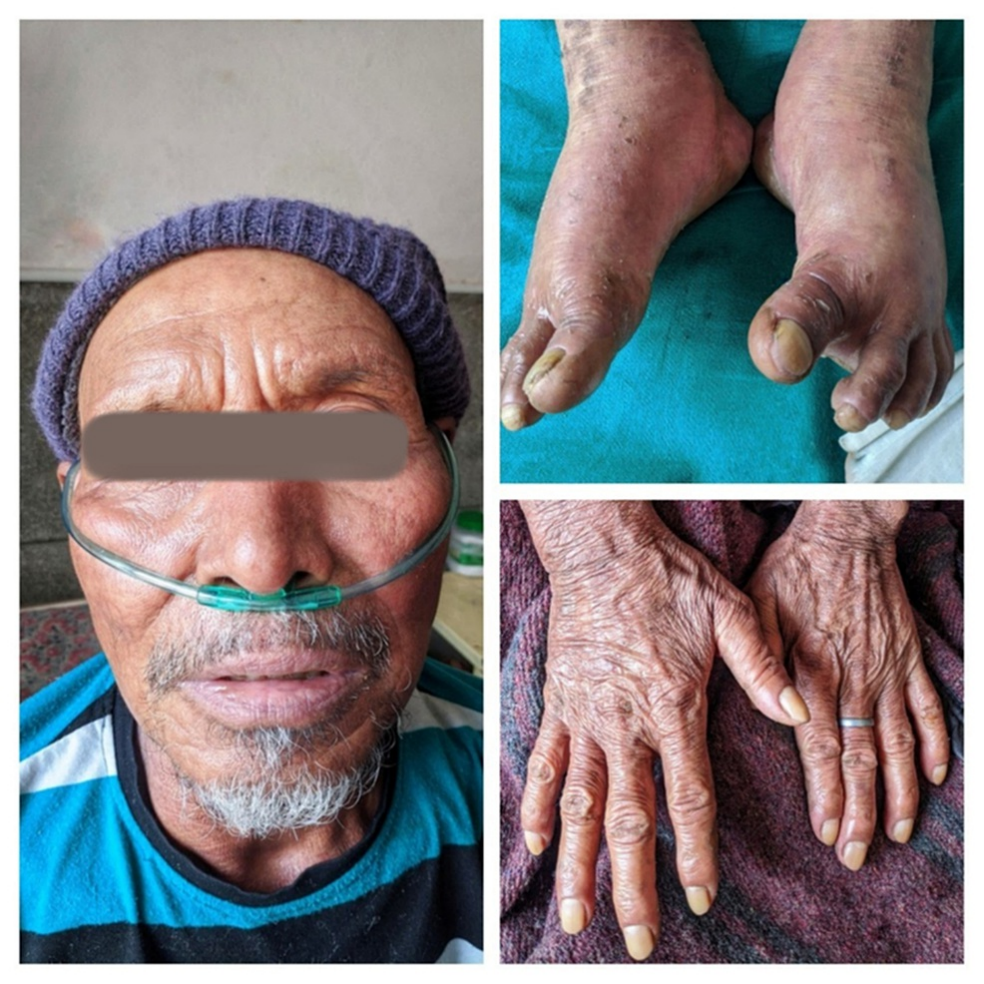
Clinical improvement and recovery

After the initial evaluation, he was put on an oral steroid, antihistaminics, topical oral antifungal suspension, and artificial tears. The patient was started on emollient to the whole body six to eight times per day. During the hospital course, the patient was improved, with desquamation of skin; after one week, his skin rash and swelling gradually recovered (Figure 4).

A rechallenge of antitubercular medication was done, according to the Philadelphia Tuberculosis Control Program [7]. The medication started initially from 1/4th dose to 2/4th on the first day in the morning and evening, respectively, and 3/4th to complete doses on the second day. Vitals and cutaneous signs were monitored every half an hour throughout the rechallenge process. Due to patient noncompliance and financial constraints, we could not continuously monitor his complete blood count, liver function, and renal function tests.

On the first day of the rechallenge, 100 mg and 200 mg of ethambutol were introduced, followed by 300 mg and 500 mg on the second day. Similarly, pyrazinamide 250 mg and 500 mg was added on the third day and was increased to 1000 mg on the fourth day. However, while rifampicin 75 mg was introduced on the fifth day, the patient complained of itching, followed by the reappearance of maculopapular rash, fever (100^o^F), and fluctuating blood pressure. As a result, the rechallenge of rifampicin was stopped. On the seventh day, the isoniazid was started with 75 mg and 150 mg, followed by 225 mg and 300 mg on the eighth day without adverse reactions. Hence, rifampicin was identified as the causative drug. The patient had then resumed ethambutol 300 mg as an alternative to rifampicin treatment, and isoniazid 800 mg for his continuation phase of ATT. On the twenty-ninth day of hospitalization, the patient was discharged with an inhaler for COPD and a tapering dose of prednisolone in addition to continuing ATT for an additional four months.

After two weeks of follow-up as an outpatient, his rash disappeared. The eosinophil levels normalized on repeat laboratory findings.

## Discussion

DRESS is a severe, potentially life-threatening adverse reaction to drugs [4]. Incidence varies by race/ethnicity because of different genetic makeup [8]⁠. The primary symptoms are rash, fever, lymphadenopathy, and eosinophilia, though these features vary from patient to patient. Multiple organs are involved such as the liver, kidney, lungs, pancreas, etc. [4,8]⁠. The pulmonary signs range from mild cough, shortness of breath, and tachypnea with chest imaging of nonspecific findings such as interstitial changes to life-threatening hypoxia and acute respiratory distress syndrome [9]⁠. The inclusion criteria and scoring system, European Registry of Severe Cutaneous Adverse Reactions to Drugs and Collection of Biological Samples (RegiSCAR) criteria for diagnosing DRESS are shown in Tables 2-3, which is most commonly used in the diagnosis of DRESS [8,10].

**Table 2:** Inclusion criteria for a potential case of HSS/DRESS in RegiSCAR HSS: hypersensitivity syndrome; DRESS: drug reaction with eosinophilia and systemic symptoms RegiSCAR: European Registry of Severe Cutaneous Adverse Reactions to Drugs and Collection of Biological Samples

Inclusion criteria for a potential case of HSS/DRESS in RegiSCAR (three or more required)
Hospitalization
Reaction suspected to be drug-related
Acute skin rash
Fever above 38 °C
Enlarged lymph nodes at least two sites Involvement of at least one internal organ
Blood count abnormalities: Lymphocytes above or below the laboratory limits, Eosinophils above the laboratory limits (in percentage or absolute count), Platelets below the laboratory limits

**Table 3:** Scoring system for the diagnosis of DRESS DRESS: drug reaction with eosinophilia and systemic symptoms; BSA: body surface area Adapted from "Variability in the clinical pattern of cutaneous side-effects of drugs with systemic symptoms: does a DRESS syndrome really exist?" by Kardaun SH, Sidoroff A, Valeyrie-Allanore L, et al. Br J Dermatol 2007; 156:609.

Clinical parameters	Score			Comments
	–1	0	1	
Fever ≥101.3°F (38.5°C)	No/unknown	unknown	Yes	
Lymphadenopathy		No/unknown	Yes	>1 cm, at least 2 sites
Eosinophilia ≥0.7 × 10^9^ or ≥10% if leucopenia		No/unknown	Yes	Score 2 points of ≥1.5 × 10^9^
Atypical lymphocytes		No/unknown	Yes	
Skin rash				
Rash suggestive of DRESS	No	Unknown	Yes	Suggestive features: ≥2 facial edemas, purpura, infiltration, desquamation
Extent ≥50% of BSA		No/unknown	Yes	
Skin biopsy suggestive of DRESS	No	Yes/unknown		
Organ involvement		No	Yes	1 point for each organ involvement (Liver, Lung, Renal, Cardiac), maximum score: 2
Disease duration ≥15 days	No/unknown	Yes		
Exclusion of other causes		No/unknown	Yes	1 point if 3 of the following tests are performed and are negative (Hepatitis A virus, Hepatitis B Virus, Hepatitis C Virus, Chlamydia, Mycoplasma, Anti-nuclear antibody, blood culture)
				If total score is <2=Excluded; 2 to 3=Possible; 4 to 5=Probable; ≥6=Definite

There is no conclusive laboratory test for DRESS; however, a total blood cell count can identify the characteristic leukocytosis with eosinophilia, and liver and renal function tests may identify internal organ involvement [1,11]⁠. A skin biopsy is not specific [11]⁠. Due to the unavailability of this service, we could not perform this test.

Th2-lymphocytes and CD8+ cells are responsible for DRESS. A type IVb hypersensitivity response is induced by Th2 cells, probably affecting the skin, while visceral organs are affected by CD8+ T cells [12]⁠. Drug-induced hypersensitivity usually occurs two to six weeks after initiation of the causative medicine [13]⁠.

DRESS following first-line anti-tubercular drug rifampicin is rare, with only a few cases reported in the literature [11]⁠. The burden of tuberculosis is high in Nepal; over 117,000 people were living with pulmonary tuberculosis, including 69,000 newly diagnosed people [14]. In addition, the proper incidence of drug reaction is not reported and presented in advanced conditions leading to life-threatening complications. However, with adequate evaluation, this condition can be managed with systemic steroids, and it has shown gratifying results in individual case reports [2,15]⁠. In addition, the withdrawal or tapering of steroids caused relapses, which suggest the efficacy of steroids in treatment. Despite this, there are no randomized clinical trials to confirm the effectiveness of the management of DRESS syndrome. Also, there is no consistency in its dosage, duration of treatment, or situations to use. DRESS syndrome improves entirely in weeks to months in most cases after causative medication withdrawal [15]⁠.

Differential diagnosis

1. Acute cutaneous lupus erythematosus: It manifests with multiple systemic involvements with acute onset of widespread rash (hallmark malar rash with sparing nasolabial fold), photosensitivity, recurrent painless oral ulcer particularly involving hard palate, arthralgia, Raynaud's phenomena, lupus hair, and laboratory abnormalities [4,16]. The biopsy shows thickened basement membrane and increased mucin. A continuous band of granular fluorescence at the dermo-epidermal junction may show by direct immunofluorescence [16].

2. Stevens-Johnson syndrome/toxic epidermal necrolysis: It presents with coalescing, purpuric macular lesion with fluid-filled vesicles or bulla (Nikolsky signs and Asboe's sign), atypical "target-like" lesions involving mucosa (ocular, oral, genital, or anal). It occurs following days to three weeks of drug exposure. In addition, it has mild eosinophilia, absence of adenopathy, tubular nephritis, and tracheobronchial necrosis [17].

3. Hypereosinophilic syndromes: It is characterized by a morbilliform eruption, urticaria, angioedema, infiltrated papules or nodules, various organs involvement without other explanation of organ damage such as the heart, gastrointestinal tract, lungs, brain, and kidneys [17].

4. Acute generalized exanthematous pustulosis: It manifests with multiple discrete sterile nonfollicular pustules of 100 to 1000 with minimal or no mucosal involvement occurs within two to three days of drugs exposure particularly involving the trunk's proximal extremities [18].

5. Drug-induced exanthems: It often presents with mild symptoms of low-grade fever, itching, and mild eosinophilia characterized as "morbilliform" (measles-like) or "rubelliform" (rubella-like) appearance with no visceral involvement. Its onset is generally from five to 14 days [19].

6. Cutaneous T-cell lymphoma: Sézary syndrome and mycosis fungoids are aggressive, leukemic variants that typically present with erythroderma and generalized lymphadenopathy. The diagnosis is based on evaluating a skin biopsy, peripheral blood with flow cytometry, and clonality studies to confirm the diagnosis [20].

## Conclusions

We report a case of a 75-year-old man with clinical features of skin exfoliation, liver and pulmonary involvement, eosinophilia, and leucocytosis, which developed eight weeks after starting anti-tuberculosis drugs. He responded well after stopping ATT and receiving a short course of steroids along with emollients. Drug rechallenge was done, maintaining proper emergency equipment and medication. DRESS recurred after rechallenging with rifampicin, which we identified as the causative drug. We also performed isoniazid, ethambutol, and pyrazinamide rechallenge, and the patient did not re-develop symptoms. The case is presented in a tuberculosis-prevalent country. Though DRESS from ATT is rare, in the context of Nepal, the prevalence and burden of disease are high. Being presented late and many differential diagnoses are possible, it is easily missed, leading to life-threatening complications without proper treatment. This report aims to provide some insight about rifampicin-induced DRESS to the primary physician in Nepal and the South Asian region, where the psychosocial impact of TB is problematic. The adverse reaction should not be neglected and should be in the differential in the initial clinical presentation. And the possibility of DRESS is considered in patients on ATT with severe rash, eosinophilia, and multiorgan involvement. Rifampicin is the first-line anti-tubercular drug, which is why ATT hypersensitivity reactions would be born in the minds of clinicians who prescribe anti-tuberculosis medications.
